# Patterns of infections and antimicrobial drugs’ prescribing among pregnant women in Saudi Arabia: a cross sectional study

**DOI:** 10.1186/s40545-020-00292-6

**Published:** 2021-01-14

**Authors:** Mohamed A. Baraka, Lina Hussain AlLehaibi, Hind Nasser AlSuwaidan, Duaa Alsulaiman, Md. Ashraful Islam, Badriyah Shadid Alotaibi, Amany Alboghdadly, Ali H. Homoud, Fuad H. Al-Ghamdi, Mastour S. Al Ghamdi, Zaheer-Ud-Din Babar

**Affiliations:** 1 Clinical Pharmacy Department, College of Pharmacy, Al Ain University, Al Ain Campus, Al Ain, United Arab Emirates; 2 Clinical Pharmacy Department, College of Pharmacy, Al-Azhar University, Cairo, Egypt; 3 First Health Cluster in Eastern Province, Dammam Medical Complex, Dammam, 32245 Saudi Arabia; 4 College of Clinical Pharmacy, Imam Abdulrahman Bin Faisal University, P.O. Box. 1982, Dammam, 31441 Saudi Arabia; 5 King Fahd Hospital of the University (KFHU), Imam Abdulrahman Bin Faisal University, Dammam, Saudi Arabia; 6 Pharmacy Practice Department, College of Clinical Pharmacy, Imam Abdulrahman Bin Faisal University, Dammam, Saudi Arabia; 7 Department of Pharmaceutical Sciences, College of Pharmacy, Princess Nourah Bint Abdulrahman University, Riyadh, Saudi Arabia; 8 College of Pharmacy, Princess Nourah Bint Abdulrahman University, Riyadh, Saudi Arabia; 9 Clinical Pharmacy Service, Johns Hopkins Aramco Healthcare, Dhahran, Saudi Arabia; 10 Pharmacy Department at Johns Hopkins Aramco Healthcare, Dhahran, Saudi Arabia; 11 Department of Pharmacology, College of Clinical Pharmacy, Imam Abdulrahman Bin Faisal University, Dammam, Saudi Arabia; 12 Department of Pharmacy, School of Applied Sciences, University of Huddersfield, Huddersfield, UK

**Keywords:** Antimicrobial resistance, Antimicrobial drugs, Infections, Antibiotics, Antimicrobial stewardship programs, Drug utilization pattern, Pregnancy, Saudi Arabia

## Abstract

**Background:**

Antimicrobial agents are among the most commonly prescribed drugs in pregnancy due to the increased susceptibility to infections during pregnancy. Antimicrobials can contribute to different maternal complications. Therefore, it is important to study their patterns in prescription and utilization. The data regarding this issue is scarce in Saudi Arabia. Therefore, the aim of this study is to generate data on the antimicrobial agents that are most commonly prescribed during pregnancy as well as their indications and safety.

**Methods:**

This is a retrospective study focusing on pregnant women with a known antimicrobial use at Johns Hopkins Aramco Healthcare (JHAH). The sample included 344 pregnant women with a total of 688 antimicrobial agents prescribed. Data was collected on the proportion of pregnant women who received antimicrobial agents and on the drug safety during pregnancy using the risk categorization system of the U.S. Food and Drug Administration (FDA).

**Results:**

The results showed that urinary tract infections (UTIs) were the most reported (59%) infectious diseases. Around 48% of pregnant women received antimicrobial medications at some point during pregnancy. The top two antimicrobial agents based on prescription frequency were B-lactams (44.6%) and azole anti-fungals (30%). The prescribed drugs in the study were found to be from classes B, C and D under the FDA risk classification system.

**Conclusion:**

The study revealed a high proportion of antimicrobials prescribed during pregnancy that might pose risks to mothers and their fetuses. Future multicenter studies are warranted to evaluate the rational prescription of antimicrobial medications during pregnancy.

## Background

Pregnancy is a critical period for women. Exposure to medications during this period might lead to adverse events that affect not only the pregnant woman but possibly the fetus [1]. Antimicrobials are commonly used among pregnant women because they are prone to different types of infections due to the lower immunity during that period [2]. On the other hand, antimicrobials remain important in reducing maternal mortality related to infections [3]. According to the published literature, the most commonly reported infections among pregnant women are respiratory tract infections, urinary tract infections and sexually transmitted infections [4, 5]. In fact, data on the use of antimicrobials in pregnancy for different indications needs to be studied to improve evidence-based care for this special population [6].

In fact, anti-infective drugs aren’t easy to deal with, since its overuse and misuse could lead to antimicrobial resistance. In 2019, WHO considered antimicrobial resistance as one of the top ten threats to global health. Therefore, all physicians and patients must be cautious while prescribing and using these medications [7, 8].

According to the Centers for Disease Control and Prevention (CDC), around 70% of women reported taking a minimum of one prescribed medication throughout their pregnancy. Amoxicillin was one of the most frequently used prescription drugs [9].

Another Omani study revealed that only 63% of prescribed antimicrobial agents were selected appropriately, and 79% of infections were treated empirically, while only 21% of patients were treated based on an obtained microorganism culture. It was also reported that 12% of empirical antimicrobials have been changed to match culture results. The most frequently prescribed antimicrobials were Piperacillin/tazobactam followed by Amoxicillin/clavulanic acid and clarithromycin [10].

Another retrospective study was conducted in an antenatal clinic in rural Ghana. The study reported that around two-thirds of pregnant women attending the clinic received antibiotic prescriptions. The most commonly prescribed antibiotics were categorized under classes B, C and D in the FDA risk classification system. The results of the study showed that 3.5% of antibacterial prescriptions were filled without proper diagnosis or justification [11].

Data on the use of medication during pregnancy in a Nepali tertiary hospital in 2016 showed an increase in the use of all drugs in the third trimester, and 12.8% of the drugs used were antimicrobials. The four most prescribed antimicrobials included Cefixime, Amoxicillin, Metronidazole and Ceftriaxone. The majority of prescribed medications were from FDA pregnancy category B [12].

In 2012, a Canadian study reported a decline in the use of broad-spectrum antibiotics over the study period from 1998 to 2002. On the other hand, the use of other classes was escalating, including macrolides, quinolones, tetracyclines, antimycotics and antimicrobials that treat urinary tract infections. Use of Penicillins and Sulfonamides was also decreasing, while Cephalosporins, anti-protozoals and antimycobacterials showed no trend. Researchers concluded that compliance with evidence-based guidelines by Canadian clinicians could be an explanation for such trends [13].

While the studies mentioned above provide valuable information on the use of antimicrobials during pregnancy, there is unfortunately scarce information on this important topic in Saudi Arabia. To the best of our knowledge, this is the first study in Saudi Arabia that comprehensively considers patterns in prescription or use of antimicrobial drugs among pregnant women.

We aim in our study to identify the most common types of infections among pregnant women in a Saudi hospital, to measure the amount of antimicrobials prescribed for pregnant women, and to assess the safety of prescribed antimicrobials during pregnancy according to FDA risk categorization.

## Methodology

### Study design and site

This is a retrospective observational study that was conducted to collect data from pregnant women with known antimicrobial utilization during pregnancy at Johns Hopkins Aramco Healthcare (JHAH), which is located in the city of Dhahran in the Eastern province of Saudi Arabia. The data was collected from patients’ electronic medical records (EMRs).

### Sampling technique and sample selection procedure

Medical records for pregnant women who had delivered their babies either through vaginal delivery or Caesarean section (C-section) as confirmed through a positive Human Chorionic Gonadotropin test (hCG) at JHAH were identified. The total number of pregnant women from December 2017 to February 2019 with a positive hCG was 5124, and all of them had been screened. A total of 2440 of these patients had received antimicrobial prescriptions, and 1760 of them had met the inclusion criteria. After collecting the medical records, we used a systematic random sampling method by selecting every fifth file. We identified 344 valid files—which was a few more than the minimum number required as per the sample size calculation—with a total number of 688 antimicrobial agents prescribed.

### Calculation of sample size

To determine the size of the sample for this study we used the power study method. This is a very useful and frequently used tool in health research for proving the adequacy of the sample size for a study. The proportion of pregnant women using antimicrobial drugs in Saudi Arabia is 3% [14]. Because the size of the population was unknown, we used the following formula to obtain an appropriate sample size:

$$n = \frac{{\left( {Z_{1 - \beta } + Z_{{{\alpha \mathord{\left/ {\vphantom {\alpha 2}} \right. \kern-\nulldelimiterspace} 2}}} } \right)^{2} \left[ {p(1 - p)} \right]}}{{d^{2} }}$$

where *n* = required sample size; *Z*_1−*β*_ = *Z* value at power 1 − *β* (minimum power 80%, value = 0.84); *Zα*_/2_ = standard normal value at a confidence level of 100 (1 − *α*) % (ideal value is 1.96 at 95% CI); *p* = referred proportion for the study 0.03 (3%); *d = *margin of error 0.05 (ideal value is 0.05 for estimated proportion in the range of 20–80%, and around 0.03 for less common or very common events [< 20% or > 80%]) [15].

Considering an 80% power of test, a 95% confidence interval, 3% marginal error, and 3% proportion rate, the formula gave us a sample size of 253.49.

In practice, may need to enroll more participants to account for potential missing/non-response errors [16]. The formula for adjusting the sample size is

$${n}_{1}=n/(1-d)$$

*n* = required sample size as per formula, *n*_1_ = adjusted sample size, *d* = the dropout rate.

Considering a 20% missing/non-response error rate, the adjusted sample size was 316.87, which is the minimum number.

### Inclusion criteria

Patients who had normal pregnancies, attended JHAH, received antimicrobial medications from December 2017 to February 2019, and delivered their babies at JHAH have been included. The age of the patients ranged between 15 and 50 years as some women may have married earlier than 18 years of age.

### Exclusion criteria

**Fig. 1:**
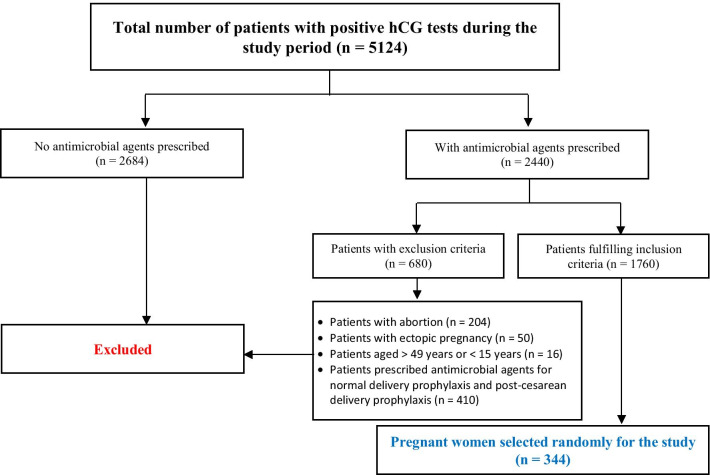
Sample selection procedures

Patients who underwent abortions, experienced ectopic pregnancies, received antimicrobials for normal delivery prophylaxis, and experienced post-cesarean delivery prophylaxis have been excluded (as shown in Fig. 1)

### Data collection

Demographic data, clinical data, anti-infective medications and comorbidities were collected for pregnant women who met the study inclusion criteria. If a patient was prescribed antimicrobial agents at any point in the pregnancy, all antimicrobial courses during the pregnancy were considered.

### Definition of the study variables

The age of the patient at the time the antimicrobial was received (gestational age) was categorized as 15–24, 25–34, 35–44, and equal to or older than 45 years.

The pregnancy trimester was calculated after the patient’s last menstrual period (LMP). Trimesters were divided as follows: first trimester (1–12 weeks), second trimester (13–27 weeks) and third trimester (28–40 weeks).

The FDA has established risk categories designated with five letters to indicate the safety of drug use during pregnancy as A, B, C, D or X. Drugs under categories A or B are considered safe for use during pregnancy. Drugs in category C could be given if benefit outweighs risk. Drugs in categories D and X are considered harmful, especially those in category X, if any, which are absolutely contraindicated.

Allergies toward drugs or food were also documented.

Drug resistance is defined as a reduction in effectiveness of antimicrobials that happens when microorganisms change after exposure to antimicrobial drugs. The resistance of a drug to a pathogen was reported by physicians in patients’ EMRs.

The mode of delivery, either vaginal or by C-section, was also recorded.

Indications of the prescribed antimicrobials, either for treatment of a known infection or as prophylaxis for pregnant women who were at high risk of infection, were also documented.

Complications that may occur during the period of pregnancy were divided into complication for preterm pregnancies (when a baby is born before 37 weeks of pregnancy) or complication for abortions. In addition, data on fetal complications—whether the fetus was exposed to any complications or abnormalities during the pregnancy—was collected. The maternal co-morbidities variable was defined as the presence of one or more additional diseases or disorders occurring with an infection. The gravida variable describes the total number of confirmed pregnancies that a patient has had, regardless of the outcome. Living children refers to children who lived beyond neonatal period. Duration of medication, expressed in days, indicates duration of treatment with antimicrobials.

Prescribed medications include antimicrobial agents used to kill or slow the growth of microbes, including antibacterial, antiviral, antifungal, and anti-parasitic drugs.

Pattern of infection designates the type of infection a pregnant woman has experienced, including bacterial, fungal, viral, and parasitic diseases.

### Data analysis

Data was analyzed using the Statistical Package for Social Sciences (IBM SPSS software version 22). The results are represented using mean and standard deviation (SD) for continuous variables and frequencies and percentages for categorical variables.

### Ethical consideration

The study received ethical approval from the university Institutional Review Board (IRB) under the following number: (IRB-UGS-2018-5-048). It was also approved by the JHAH IRB under (IRB number 18-07).

## Results

**Fig. 2:**
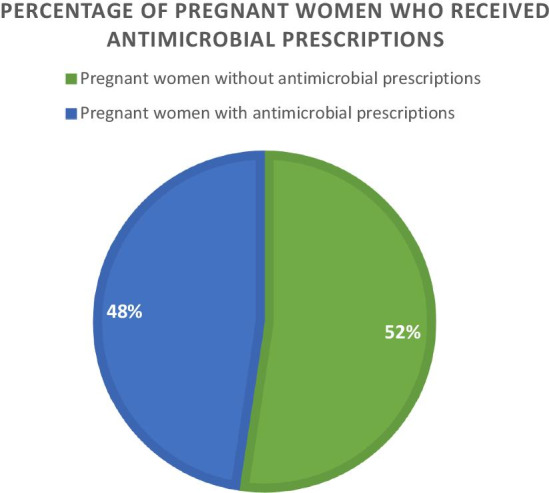
Percentage of pregnant women who received antimicrobial prescriptions

Our study revealed that 48% of pregnant women received antimicrobial prescriptions (Fig. 2). The mean age of the respondents was 19.19 (SD 6.33) years, ranging from 17 to 48 years. More than half (55.5%) of the respondents were 25–34 years old. However, the mean gestational age was 23.49 weeks (SD 10.35) as shown in Tables 1a and 2.

**Table 1:** **a** Baseline characteristics of study participants/pregnant women (*n* = 344); **b** Baseline characteristics of study variables related to prescribed antimicrobials (*n* = 688)

Variables	Group	Frequency (*n*)	Percentage (%)
(a)			
Age	15–24	77	22.4
	25–34	191	55.5
	35–44	73	21.3
	≥ 45	3	0.9
Drug resistance (*n* = 324)	No	272	84.0
	Yes	52	16.0
	B-lactam	20	38.5
	MDR	17	32.7
	Quinolones	15	28.8
Mode of delivery	Vaginal delivery	245	71.2
	C-section	99	28.7
Mother comorbidities	No	198	56.9
	Yes	146	42.4
Maternal complications	Abortions	205	29.8
	Preterm pregnancies	152	22.1
Fetal complications	No	297	86.3
	Yes	47	13.6
(b)			
Antimicrobial use per trimester (*n* = 685)	First	148	21.6
	Second	212	30.9
	Third	325	47.4
FDA category for safety drug use during pregnancy	Category A	0	0
	Category B	457	66.4
	Category C	224	32.6
	Category D	7	1.0
	Category X	0	0
Allergy	No	633	92.0
	Yes	55	8.0
	B-lactam	12	21.8
	Dopaminergic	11	20.0
	Quinolones	7	12.7
	NSAIDs	6	10.9
	Food allergy	6	10.9
	Danozol	3	5.5
	Multi drug allergy	3	5.5
	Corticosteroids	2	3.6
	Tetracycline	2	3.6
	Miscellaneous	2	3.6
	Sulfa drug	1	1.8
Indication of antibiotic use	Treatment	681	99.0
	Prophylaxis	7	1.0

**Table 2:** Mean, standard deviation (SD), 95% confidence interval (CI) of mean and range for some study variables

Variables	Mean ± SD or median (IQR)	95% CI of mean	Range
Age (years)	19.19 ± 6.33	L-28.72, U-29.67	17 to 48
Gestational age (week)	23.49 ± 10.35	L-22.71, U-24.27	1 to 41
Gravida	3.12 ± 2.37	L-2.94, U-3.30	1 to 13
Number of term of pregnancies	2.17 ± 1.77	L-2.03, U-2.30	0 to 8
Number of living children (L)	2.36 ± 1.78	L-2.23, U-2.50	0 to 8
Duration of taking medicine (days)	7.0 (2.0)	–	1 to 120

The majority of patients did not experience any bacterial resistance (84.0%). There were 20 missing data for the drug resistance variable. According to the susceptibility tests that were obtained with cultures, microorganisms were most likely resistant to B-lactam antibiotics (38.5%), followed by multi-drug resistance (MDR) (32.7%) as shown in Table 1a.

Around three quarters of participating pregnant women had completed vaginal delivery (71.2%). Gravida mean ± SD was 3.12 ± 2.37 with a range of 1 to 13 pregnancies as shown in Tables 1a and 2.

Around half of the mothers were following up after their pregnancies without any comorbidities (56.9%). Complications experienced by pregnant women were mainly abortions (29.8%). Most of the babies had no complications after their mothers received antimicrobial drugs (86.3%). Mean ± SD for the number of living children was 2.36 ± 1.78 as shown in Tables 1a and 2.

Around half of the antimicrobial prescriptions were issued during the third trimester (47.4%). The lowest percentage of antimicrobials prescribed was during the first trimester (21.6%). Three antimicrobial agents did not specify the trimester, as shown in Table 1b.

FDA categorization for safety of drug use during pregnancy fell into classes B, C and D. However, antimicrobials in classes A and X were not prescribed. Most of the drugs fell into class B (66.4%), as shown in Table 1b.

The vast majority of patients had no known allergies (NKA) (92.0%) to the prescribed drugs. Beta lactams antibiotics accounted for (21.8%) of active drug allergies among pregnant women, followed by dopaminergic drugs and quinolones with (20.0%) and (12.7%) respectively, as shown in Table 1b.

Most of the pregnant women were prescribed antimicrobials to treat infections (99.0%), and only (1.0%) were prescribed as prophylaxis. Median duration for taking an antimicrobial was 7.0 (2.0) with a range of 1–120 days, as shown in Tables 1b and 2.

Out of 5124 pregnant women who attended the hospital between December 2017 and February 2019, 2440 were exposed to antimicrobials for different indications, and 2684 women were not exposed.

### Pattern of infections affecting pregnant women

The majority of patients in this study suffered from bacterial infections (64.0%). UTI from bacterial infection had the highest proportion (59.3%) followed by fungal infections (34.5%). There were 101 missing data as shown in Table 3.

**Table 3:** Patterns of infection among pregnant women from the Eastern region, Saudi Arabia (*n* = 587)

Variables	Group	Frequency (*n*)	Percentage (%)
Pattern of infection	Bacterial infection	376	64.0
	UTIs	223	59.3
	URTIs	72	19.1
	Skin and soft-tissue infection	36	9.5
	Intra-abdominal infection	24	6.3
	LRTIs	11	2.9
	Eye infection	10	2.6
	Fungal infection	203	34.5
	Parasitic infection	4	0.6
	Viral infection	4	0.6

### Patterns of antimicrobial prescriptions among pregnant women including systemic and/or topical routes

The most frequent antimicrobial prescriptions among pregnant women were B-lactams (44.6%) followed by prescriptions of azoles (30.2%). The other antimicrobial prescriptions were not as frequent; these included macrolides (7.7%), quinolones (6.7%) and other antibiotics (6.5%), as shown in Table 4.

**Table 4:** Pattern of prescribed antimicrobials among pregnant women from the Eastern region of Saudi Arabia (*n* = 688)

Variables	Group	Frequency (*n*)	Percentage (%)
Prescribed medication	B-lactams	307	44.6
	Azoles	208	30.2
	Macrolides	53	7.7
	Quinolones	46	6.7
	Other antibiotics	45	6.5
	Aminoglycosides	11	1.6
	Antivirals	6	0.9
	Other anti-fungals	6	0.9
	Antimalarials	5	0.7
	Tetracyclines	1	0.1

More than half of the patients who have been prescribed antimicrobials, or 55.8%, were treated empirically. Microbiological cultures were requested for the remaining 44.2%, of which 12.5% revealed no growth. *Escherichia coli* bacteria were identified in 8.7% of performed cultures followed by mixed flora with 8.4%. Other pathogens were *Streptococcus* *agalactiae* with 3.5%, *Klebsiella pneumoniae* with 2.9%, *Candida* *albicans* with 2.7%, Extended-Spectrum Beta-Lactamase (ESBL)-producing *E. coli* with 1.1%, and *Staphylococcus aureus* with 0.9%. There were 32 missing data in this part. The details of these microbiological cultures are described in Table 5.

**Table 5:** Descriptive statistics for microbiological culture among pregnant mothers from the Eastern region of Saudi Arabia (*n* = 656)

Variables	Group	Frequency (*n*)	Percentage (%)
Microbiological culture	No isolate	366	55.8
	No growth	82	12.5
	*Escherichia coli*	57	8.7
	Mixed flora	55	8.4
	*Streptococcus agalactiae*	23	3.5
	*Klebsiella pneumoniae*	19	2.9
	*Candida albicans*	18	2.7
	ESBL *E. coli*	7	1.1
	*Staphylococcus aureus*	6	0.9
	*Acinetobacter baumannii* complex	3	0.5
	*Candida glabrata*	3	0.5
	*Lactobacillus* species	3	0.5
	*Campylobacter jejuni*	2	0.3
	*Enterobacter aerogenes*	2	0.3
	*Enterobacter cloacae*	2	0.3
	*Enterococcus faecalis*	2	0.3
	*Staphylococcus epidermidis*	2	0.3
	*Diphtheroid bacilli*	1	0.2
	*Helicobacter pylori*	1	0.2
	*Pseudomonas aeruginosa*	1	0.2
	*Staphylococcus haemolyticus*	1	0.2
	Total	656	100.0

## Discussion

### Indications for antimicrobial use

The most prevalent infectious diseases among pregnant women in JHAH were bacterial infections (predominantly UTI and RTIs) and fungal infections. Our findings conformed with the Canadian study as RTIs and UTIs were the most prevalent bacterial infections in both studies. However, RTIs ranked first in the Canadian study, whereas UTIs had the highest proportion in our study. This may be due to the differences in weather between the two countries. The weather in Canada may contribute to a higher prevalence of RTIs because of the long, harsh winters as mentioned in their study [17]. In Saudi Arabia, meanwhile, winter is much shorter and warmer. The general prevalence of UTIs in pregnant women may be due to the physiological changes that arise in the gestational period, when the uterus grows and blocks the drainage of urine from the bladder, thus creating a susceptible medium for infections [18].

The second most common microbial infection at JHAH was fungal. This can be explained by the physiological decline in immunity in addition to the hormonal fluctuations during pregnancy [2, 19].

### Percentage of antimicrobial exposure

Forty-eight percent of pregnant women in our study received antimicrobial medication during their pregnancies. This was higher than the prevalence of anti-infective use reported in a study conducted in 2012 in Quebec, Canada [13]. However, it was less than the documented antibiotic use in a recent study conducted in an antenatal clinic in rural Ghana [11].

### Classes of prescribed antimicrobials

Top two antimicrobial agents based on prescription frequency were B-lactams and azole antifungals. This conformed with the findings of the study conducted in Quebec, Canada, where Penicillins was the most prescribed antimicrobial class, while the next top three antimicrobials were macrolides, quinolones and antifungal agents respectively [13]. This is also in agreement with the findings of the Ghanaian study, where beta lactam antibiotics—i.e., Cephalosporins and Penicillins—also represented the majority of antibiotics used [11], and as reported in another study conducted in a hospital in Western Nepal [12]. The superiority of beta lactams at JHAH may be linked to the high number of UTI infections. Beta lactams are recommended for use during pregnancy to treat Asymptomatic Bacteriuria and UTIs [20]. Moreover, the frequent prescriptions of azole antifungals were related to the frequent fungal infections prevalent among pregnant women at JHAH. Based on FDA risk categorization, Penicillins and Cephalosporins are considered safe options in pregnancy. If used systemically, Azoles could be teratogenic in animals and humans. However, topical azoles are not absorbed, or are minimally absorbed, and hence are permitted at any stage of pregnancy [21, 22]. In our data, the prescription of azoles for pregnant women was systemic and topical.

### Pregnancy risk categories

The majority of antimicrobial drugs prescribed to our participants belong to FDA category B (66.4%), followed by category C (32.6%) and category D (1.0%), which is considered harmful according to FDA recommendations. No antimicrobials from category A or X were documented in our study. Similar findings were revealed in the study conducted in an antenatal clinic in rural Ghana between 2011 and 2015, where the antimicrobials taken by pregnant women were mainly from FDA category B (69.6%), with fewer drugs prescribed from categories C (2.9%) and D (0.5%). Antimicrobials in categories A and X were not prescribed in the Ghanaian study [11]. In addition to the risks categorized by the FDA, some cases of exposure to potentially harmful drugs prescribed inappropriately against therapeutic guidelines have been identified. For example, one of the pregnant women was diagnosed with acne excoriee in the first trimester and received Minocycline 100 mg orally twice daily for 2 weeks. Tetracycline antibiotics (including doxycycline and minocycline) are known to exert toxic effects on fetal teeth and bones as they bind to calcium orthophosphate and undergo active deposition in teeth and bones of the fetuses. It has been documented that oral antibiotics such as Erythromycin, Azithromycin, Cephalexin and Amoxicillin are more appropriate for treating acne during pregnancy [23]. In another example, a pregnant woman in her first trimester had a diagnosis of UTI caused by Candida and received Fluconazole 200 mg orally once daily for 1 week. A recent study reported that the use of oral fluconazole in the first trimester is associated with musculoskeletal malformations, and the researchers recommended the use of topical azoles as an alternative treatment [24].

### Drug use per trimester

In our study, antimicrobials were prescribed in all three trimesters with more frequent prescription in the third trimester. A similar study shows total medication use during pregnancy was maximum in the third trimester with an average of (3.88) drugs per patient, followed by the second trimester with (3.05) drugs per patient and the first trimester with (3.01) drugs per patient [12]. These data might be explained by the general perception that there is little risk for development of major malformation in the fetus beyond the organogenesis phase in the first trimester [25, 26]. For this reason, physicians in our institution might have been more comfortable prescribing medications in the third trimester. Another study [27] revealed that the prevalence of prescribed medications was higher in the first trimester (47.0%), which is considered to be the critical period for most major congenital abnormalities [28]. However, at JHAH, most study participants who received anti-infective medication in the first trimester did so before confirming pregnancy.

### Microbiological culture and empiric treatment

Almost half of the patients had microbiological cultures prior to the initiation of antimicrobial agents, which revealed negative results in 12.5% of the cultures. However, in the positive cultures, the most predominant microorganism was *Escherichia coli*, followed by *Streptococcus agalactiae*, *Klebsiella pneumoniae*, *Candida albicans*, ESBL *E. coli* and *Staphylococcus aureus*. Al Yamani et al. reported similar results in which the most common organisms in their hospital were gram-negative bacteria (*E. coli*, followed by *Klebsiella pneumoniae*, *Pseudomonas aeruginosa*, methicillin-resistant *Staphylococcus aureus* [MRSA], and Acinetobacter). In their institution, the practice differed in obtaining the cultures before the antimicrobial course initiation, and cultures were collected from only one-quarter of their patients [10]. This may reflect the attitude of JHAH practitioners in considering the bacterial culture and its importance before prescribing antimicrobials as availability of pathogens and antimicrobial susceptibility testing can be helpful for antimicrobial stewardship programs [29].

### Clinical and policy impact of the study

The present study describes the overall practices of prescribing antimicrobial agents in pregnant women and the most common types of infectious diseases occurring during pregnancy in a Saudi Arabian hospital. These findings are expected to help in generating knowledge about better utilization of antimicrobials for pregnant women, thereby improving the prescription of antimicrobials for pregnant women through safe selection of antimicrobial regimens. They may also shed light on the prescription patterns of different antimicrobials among pregnant women. Awareness and educational programs are warranted to help healthcare providers rationalize prescription of antimicrobials for pregnant women.

### Study limitations and recommendations for future research

The current study has several limitations. First, prescription of antimicrobials during pregnancy was evaluated in a single center, and every antimicrobial prescription was considered an encounter and was counted as a separate file for statistical purposes. Therefore, caution is required for generalizing the findings to the entire population. Second, the poor documentation in some encounters has led to a lack of information regarding treatment indications. Therefore, we could not explain or connect the use of antimicrobials to the disease state or maternal and fetal consequences nor to check appropriateness of antimicrobials prescribing against published guidelines. Moreover, this resulted in missing data that hindered the correlation of antimicrobial use with demographic data. Nevertheless, the study investigated the prescription patterns of antimicrobial agents during pregnancy. It highlighted the general prescription practices and most common infections at the JHAH hospital in the city of Dhahran.

## Conclusion

Our study revealed a high frequency of antimicrobials prescribed during pregnancy that might pose risks to mothers and their fetuses. Different approaches are needed to increase awareness among healthcare providers as well as pregnant women about the common types of infections during pregnancy and how to prevent them. The study has identified a gap in training and a need for educational programs to avoid prescribing antimicrobials in FDA categories C and D unless well indicated and benefits outweigh risks. Further studies are warranted in order to identify factors associated with such antimicrobial prescription and to generalize the results to the rest of the population.

## Data Availability

All data and materials are available upon request to any scientist wishing to use them.

## Abbreviations

JHAH: Johns Hopkins Aramco Healthcare
UTIs: Urinary tract infections
RTIs: Respiratory tract infections
URTIs: Upper respiratory tract infections
LRTIs: Lower respiratory tract infections
C-section: Caesarean section
FDA: Food and Drug Administration
EMRs: Electronic medical records
hCG: Human Chorionic Gonadotropin
LMP: Last menstrual period
SPSS: Statistical Package for Social Sciences
IRB: Institutional Review Board
SD: Standard deviation
IQR: Interquartile range
CI: Confidence interval
NSAIDs: Non-steroidal anti-inflammatory drugs
NKA: No known allergy
MDR: Multi-drug resistance
MRSA: Methicillin-resistant *Staphylococcus aureus*
ESBL *E. coli*: Extended-Spectrum Beta-Lactamase producing *E. coli*
*E. coli*: *Escherichia coli*
